# Effects of the Cathepsin K Inhibitor ONO-5334 and Concomitant Use of ONO-5334 with Methotrexate on Collagen-Induced Arthritis in Cynomolgus Monkeys

**DOI:** 10.1155/2019/5710340

**Published:** 2019-02-17

**Authors:** Hiroyuki Yamada, Hiroshi Mori, Yasutomo Nakanishi, Satoshi Nishikawa, Yasuaki Hashimoto, Yasuo Ochi, Makoto Tanaka, Kazuhito Kawabata

**Affiliations:** Discovery Research Laboratories, Ono Pharmaceutical Co., Ltd., 3-1-1 Sakurai, Shimamoto-cho, Mishima-gun, Osaka 618-8585, Japan

## Abstract

We examined whether the cathepsin K inhibitor, ONO-5334, administered alone or in combination with methotrexate (MTX), could ameliorate joint destruction evoked by collagen-induced arthritis (CIA) in female cynomolgus monkeys. CIA was induced by immunizing with bovine type II collagen. ONO-5334 (30 mg/kg/day) was orally administered once daily and MTX (10 mg/body/day) twice weekly for 9 weeks. X-ray (evaluation of joint destruction) and swelling (inflammatory) scores of proximal interphalangeal (PIP), distal interphalangeal (DIP), and metacarpophalangeal (MP) joints were evaluated. Urinary concentrations of C-terminal telopeptide of type I collagen (CTX-I) and type II collagen (CTX-II) were measured. Arthritis, accompanied by bone and cartilage destruction, was successfully induced in this collagen-induced arthritis monkey model. ONO-5334 showed no suppressive effect on joint swelling, while the joint swelling scores in the MTX and combination (ONO-5334 + MTX) groups were less than 50% compared with the control group. ONO-5334 decreased X-ray score by a mean of 64% (p<0.05 vs the control group), and MTX also decreased in X-ray score by a mean of 46% but with no statistical significance. Combination of ONO-5334 and MTX further decreased the X-ray score by 28% over MTX group (74% reduction vs the control group, p<0.01). Maximum increase in CTX-I (10-fold) and CTX-II (7-fold) compared to baseline was observed at 7 and 3 weeks after the first sensitization, respectively. After treatment with ONO-5334 alone or in combination with MTX, concentrations were maintained near baseline for both markers. In conclusion, ONO-5334 prevented joint destruction but not joint inflammation in this monkey CIA model. Concomitant use of ONO-5334 with MTX reduced architectural joint destruction compared to MTX alone; therefore, ONO-5334 may help to prevent joint destruction in combination with MTX for the treatment of rheumatoid arthritis.

## 1. Introduction

Rheumatoid arthritis (RA) is an autoimmune disease that causes chronic inflammation of the joints and can also cause inflammation of the tissue around the joints, as well as other organs in the body [1]. Joint inflammation in RA causes swelling, pain, stiffness, and redness in the joints. The chronic inflammation also leads to the destruction of cartilage, bone, and ligaments causing deformity of the joints. Damage to the joints can occur early in the disease and be progressive. Radiographic changes occur within 2 years of disease onset in 50-70% of RA patients and are considered related to the functional disability. Methotrexate (MTX) is the most common disease modifying antirheumatic drug (DMARD) used in the management of RA [2, 3]. MTX can be prescribed alone or in combination with other agents and is considered the gold standard in the management of RA [4]. However, MTX is not always efficacious and can induce significant adverse events (e.g., bone marrow suppression, hepatotoxicity, etc.) in patients [5]. Biologic DMARDs such as tumor necrosis factor (TNF) blocking agents, anti-interleukin-6 (IL-6) receptor antibody, anti-CD20 antibody, and CTLA4-Ig have significant positive effects on both signs and symptoms and inhibition of structural damage but have a high cost, safety concerns (e.g., infection, allergy, etc.), and inconvenience of intravenous or subcutaneous injection. Thus, new treatment agents for the inhibition of joint destruction are considered important to preserve functional ability.

Cathepsin K is a proteolytic enzyme that can degrade triple helix of type I and II collagens and other components, such as osteonectin and aggrecan, of the extracellular matrix in bone and cartilage [6, 7]. Cathepsin K is highly expressed by osteoclasts [8–10] and plays a central role in bone resorption. It has been also demonstrated that cathepsin K is expressed in synovial fibroblasts and macrophages in rheumatoid arthritic joints and osteoarthritic cartilage [11–14]. In addition, overexpression of cathepsin K in transgenic mice leads to the development of synovitis and cartilage degeneration [15], and inhibition of cathepsin K reduced bone erosion, cartilage degradation, and inflammation in a mice model of collagen-induced arthritis (CIA) [16]. Therefore, it is conceivable that cathepsin K could be a beneficial therapeutic target for joint destruction in RA.

ONO-5334 is a low molecular weight synthetic inhibitor of cathepsin K with Ki value of 0.1 nM [17]. In previous studies, we have shown that ONO-5334 suppresses bone resorption and increases bone mineral density in ovariectomized (OVX) cynomolgus monkeys [18, 19] and in patients with postmenopausal osteoporosis [20, 21]. The monkey CIA model may be more suitable for prediction of clinical efficacy in RA than rodent CIA models because monkeys are phylogenetically and physiologically close to human beings [22, 23]. In this study, we evaluated the preventive effects of ONO-5334 alone or in combination with MTX on joint swelling and destruction in CIA monkeys.

## 2. Materials and Methods

### 2.1. Compounds

ONO-5334, N-((1S)-3-{(2Z)-2-[(4R)-3,4-dimethyl-1,3-thiazolidin-2-ylidene] hydrazino}-2,3-dioxo-1-(tetrahydro-2H-pyran-4-yl) propyl) cycloheptanecarboxamide, was synthesized in our laboratories (Ono Pharmaceutical Co., Ltd., Osaka, Japan). MTX was purchased from Wako Pure Chemical Industries, Ltd., Osaka, Japan.

### 2.2. Animal Experiments

All animal experiments were approved by the Institutional Animal Care and Use Committee of Shin Nippon Biomedical Laboratories, Ltd. (Kagoshima, Japan), and performed in accordance with the ethics criteria contained in the bylaws of the committee. Thirty-six adult female cynomolgus macaques (Macaca fascicularis) weighing 2.25–3.00 kg, aged between 3 and 6 years, were obtained from four suppliers, Gaoyao Kangda Laboratory Animals Science and Technology Co., Ltd. (Guangdong, China), Guangzhou Kesen Imports and Exports Co., Ltd. (Guangzhou, China), Guangxi Grandforest Scientific Primate Co., Ltd. (Guangxi, China), and Guangdong Scientific Instruments and Material Import/Export Corporation (Guangzhou, China), and were housed individually in a room maintained at a temperature of 24.2–26.4°C and a relative humidity of 46–65%, under a 12-hour light/dark cycle. Each animal was provided with approximately 108g of pellet food (Purina Mills, LLC, MO, USA) once daily and were given free accessed to tap water. Based on their body weights, animals were allocated by randomization to the following four groups (9 animals per group): control, ONO-5334, MTX, or combination (ONO-5334 + MTX) group.

### 2.3. Induction of Collagen-Induced Arthritis

Arthritis was induced by immunizing with bovine type II collagen in the presence of Freund's complete adjuvant as reported previously [24]. In summary, bovine type II collagen (4 mg/mL, Collagen Research Center, Tokyo, Japan) was emulsified in an equal volume of Freund's complete adjuvant (Becton Dickinson, Grayson, GA, USA) and maintained on ice until use. Each animal was administered intradermally with 2 mL/body of emulsion by 19 injections on the back and one injection on the base of the tail (the first sensitization), and the second sensitization was performed 3 weeks after the first sensitization in the same manner.

### 2.4. Test Article Administration

ONO-5334 was suspended in 0.5% methylcellulose (MC) solution, and 10 mg of MTX was put into a gelatin capsule (size #2, Noichi K.K., Japan). Control group was orally given 0.5% MC once daily and an empty capsule twice weekly. ONO-5334 and combination groups were orally given ONO-5334 at dose of 30 mg/kg once daily. MTX and combination groups were orally given a capsule containing 10 mg of MTX twice weekly. ONO-5334 and MTX groups were also given empty capsule twice weekly and 0.5% MC once daily, respectively. Animals received test articles for 9 weeks from the day of the first sensitization. The dose level of ONO-5334 was decided based on the results from an OVX monkey osteoporosis study [18]. ONO-5334 at 30 mg/kg prevented OVX-induced decreases in bone mass and strength. Regarding the dose level of MTX, in a preliminary study of a monkey CIA model MTX diminished phalangeal joint swelling at 10 mg/body twice weekly [25].

### 2.5. Assessment of General Condition

The general condition of animals was evaluated before the first sensitization and on 3, 5, 7, and 9 weeks after the first sensitization in accordance with the following criteria: score 0: no abnormality; score 1: difficulty in hanging from the bars of the home cage by the fingers; score 2: inability to hang from the bars of the home cage by the fingers (using wrist); score 3: movement only by using forelimbs or hindlimbs; score 4: crouching.

### 2.6. Assessment of Joint Swelling

The joint swelling level was evaluated before the first sensitization and at 3, 5, 7, and 9 weeks after the first sensitization. Animals were anaesthetized by intramuscular injection of 50 mg/mL of ketamine hydrochloride (0.2 mL/kg, Kamud Drugs Pvt. Ltd., Maharashtra, India). The swelling levels of the distal interphalangeal (DIP), proximal interphalangeal (PIP), and metacarpophalangeal (MP) joints in 5 digits (in the first digit: interphalangeal and MP joints) of the fore and hind limbs as well as wrist, ankle, elbow, and knee joints in individual animals (total 64 joints/animal) were evaluated in a blinded manner in accordance with the following criteria: score 0: no abnormality; score 1: swelling not visible but can be determined by touch; score 2: swelling slightly visible and can be confirmed by touch; score 3: swelling clearly visible; score 4: rigidity of the joints [25]. The joint swelling score for each animal was defined as the total score of individual joints (arthritic score of 0–4, total 64 joints/body, maximum score 256).

### 2.7. X-Ray Examinations

Animals were examined under anaesthesia by intramuscular injection of ketamine hydrochloride as described above, with a diagnostic X-ray system (DREX-WIN64, Toshiba Medical Systems Corporation, Japan) before the first sensitization and 9 weeks after the first sensitization. The severity of destruction (no abnormality, mild: joint space narrowing, bone atrophy, joint space narrowing + bone atrophy, severe: bone erosion, architectural joint destruction) in each joint including DIP, PIP, and MP joints in the second, third, fourth, and fifth digits of the forelimbs and hindlimbs (total 48 joints/animal) was determined by X-ray image in a blinded manner [25]. The X-ray score for each animal was defined as the total number of joints in which abnormal changes were observed (maximum score: 48). The percentage number of joints by severity of destruction (6 findings) in all evaluated joints of each group was calculated. This calculation was also conducted by DIP, PIP, or MP joints. The total number of all evaluated joints in the control and MTX groups was 384 (48 joints/animal, 8 animals) and in the ONO-5334 and combination groups was 432 (48 joints/animal, 9 animals). The total number of evaluated DIP, PIP, or MP joints in the control and MTX groups was 128 (16 joints/animal, 8 animals) and in the ONO-5334 and combination groups was 144 (16 joints/9 animals).

### 2.8. Bone and Cartilage Turnover Markers in Urine

Twenty-four-hour cumulated urine samples were collected by attaching trays to the cages before the first sensitization and at 3, 5, 7, and 9 weeks after the first sensitization. Urine samples were centrifuged and the supernatants were stored at -70°C. The bone resorption marker, C-terminal telopeptide of type I collagen (CTX-I) and the cartilage degradation marker, and C-terminal telopeptide of type II collagen (CTX-II) were measured by using Urine CrossLaps ELISA (IDS, Boldon, UK) and Preclinical CartiLaps ELISA (IDS, Boldon, UK), respectively. Urinary CTX-I and CTX-II data were normalized to creatinine concentration.

### 2.9. Statistical Analysis

Statistical analyses were performed using SAS 9.1 (SAS Institute Japan Ltd.). General condition score and joint swelling score were analysed among the control, ONO-5334, MTX, and combination groups using the Steel Dwass test adjusting for a multiplicity. X-ray score, urinary CTX-I, and CTX-II concentration were analysed among all groups using Tukey test adjusting for a multiplicity. A correlation analysis between joint swelling and X-ray scores was performed using Spearman correlation test. Data obtained from two animals, one animal in the control group and another in the MTX group, were excluded because of nonrelated morbidity during the study period.

## 3. Results

### 3.1. General Condition Scores

General condition score increased almost time dependently in the control group (Table 1). During the dosing period, one animal in the control group, four animals in the ONO-5334 group, six animals in the MTX group, and four animals in the combination group had no abnormality. The mean general condition scores in the ONO-5334, MTX, and combination groups were slightly lower than that in the control group, but there were no significant differences among groups.

### 3.2. Joint Swelling Scores

**Figure 1:**
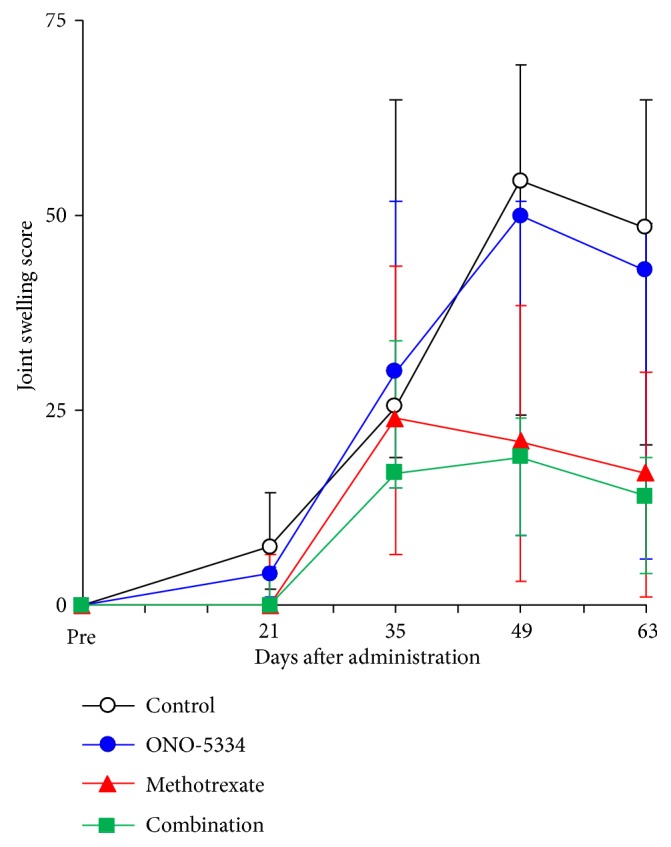
Joint swelling score in collagen-induced arthritis monkeys. The swelling levels of DIP, PIP, and MP joints in 5 digits of the forelimbs and hindlimbs as well as wrist, ankle, elbow, and knee joints in individual animals were evaluated in a blinded manner in accordance with the following criteria: score 0: no abnormality; score 1: swelling not visible but can be determined by touch; score 2: swelling slightly visible and can be confirmed by touch; score 3: swelling clearly visible; score 4: rigidity of the joints. The maximum score is 256 (total 64 joints/body and arthritic scores 0–4). Data are expressed as median (quantile 3-1); control (n=8), ONO-5334 (n=9), methotrexate (n=8), and combination (n=9).

Joint swelling in the control group increased with time until 7 weeks (Figure 1). While ONO-5334 alone did not diminish joint swelling, MTX and combination of MTX and ONO-5334 appeared to equally decrease joint swelling. Median joint swelling scores in the MTX and combination groups were less than 50% compared with the control group at 7 and 9 weeks, but there were no significant differences.

### 3.3. X-Ray Examinations

**Figure 2:**
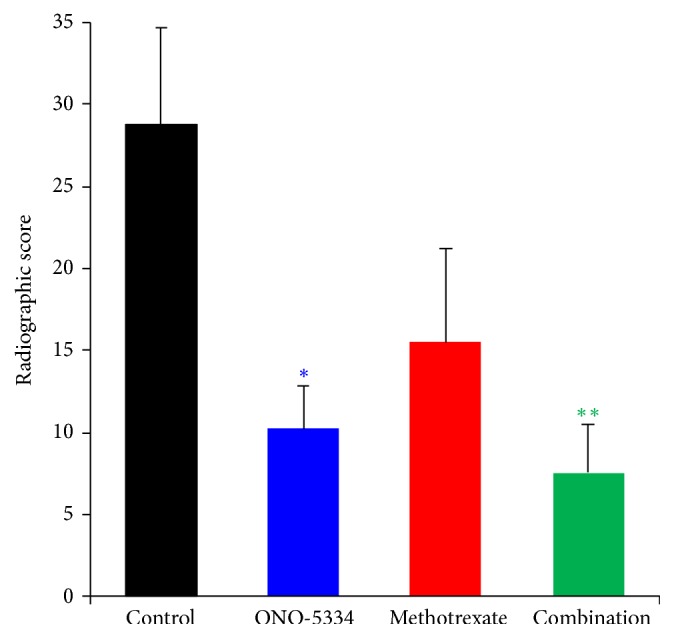
X-ray examination of total affected joint number (DIP, PIP, and MP joints in the second, third, fourth, and fifth digits of the forelimbs and hindlimbs: total 48 joints/monkey) in collagen-induced arthritis monkeys. Data are expressed as mean ± SE of control (n=8), ONO-5334 (n=9), methotrexate (n=8), and combination (n=9) groups. *∗*p<0.05 and *∗∗*p<0.01 versus control group (Tukey test).

There are no abnormal X-ray findings in any treatment group before the first sensitization. Nine weeks after the first sensitization, ONO-5334 decreased X-ray scores by a mean of 64% (p<0.05 vs the control group), and MTX decreased X-ray scores by a mean of 46% but there was no statistical significance (Figure 2). A combination of ONO-5334 and MTX decreased X-ray scores by a mean of 74% (p<0.01 vs the control group).

**Figure 3:**
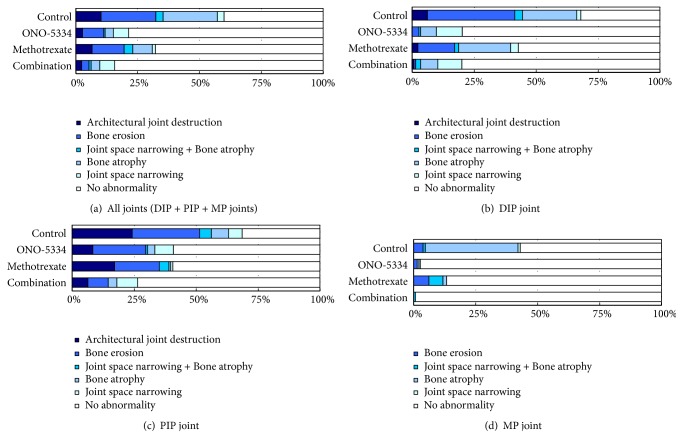
Severity of joint destruction at (a) all, (b) DIP, (c) PIP, and (d) MP joints in collagen-induced arthritis monkeys. The number of all joints (DIP + PIP + MP) was 384 (48 joints/monkey, n=8) in the control and MTX group and 432 (48 joints/monkey, n=9) in the ONO-5334 and ONO-5334/MTX groups.

The severity of destruction in the phalangeal joints was compared among treatment groups (Figure 3). The percentage number of joints with no abnormality in all evaluated joints was higher in the ONO-5334 groups compared with the control group (79% vs 40%) and was higher in the combination group than in the MTX group (84% vs 68%). Conversely, the percentage number of joints with severe destruction was lower in the ONO-5334 group compared with the control group (11% vs 32%) and was lower in the combination group than in the MTX group (5% vs 20%) (Figure 3(a)). Separate analyses by DIP, PIP, or MP joint alone revealed that severity of destruction was different among these joint sites (Figures 3(b)–3(d)). The percentage number of joints with severe destruction in the control group was much higher in DIP and PIP than in MP joints. In DIP, PIP and MP joints, ONO-5334 decreased the percentage number of joints with severe destruction compared with the control group, and the combination of ONO-5334 and MTX also decreased this percentage compared to MTX alone.

### 3.4. Relationship between Joint Swelling Score and X-Ray Score

**Figure 4:**
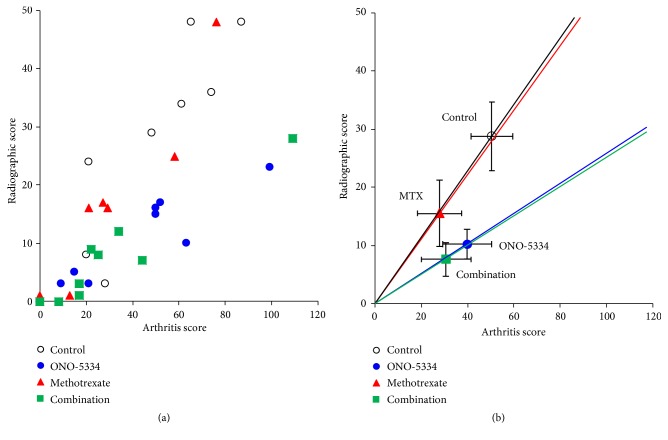
Relation between joint swelling score at peak and X-ray score at day 63 for (a) individual animals and (b) treatment groups in collagen-induced arthritis monkeys. Data are expressed as mean ± SE; control (n=8), ONO-5334 (n=9), methotrexate (n=8), and combination (n=9).

A statistically significant correlation was observed between joint swelling score at peak and X-ray score at day 63 in each group (correlation coefficient, p value); control group (0.89, p<0.01), ONO-5334 group (0.87, p<0.01), MTX group (0.94, p<0.01), and combination group (0.87, p<0.01). A linear relationship between joint swelling and X-ray scores was observed in each group on the scatter plot using all animal data; however, the distribution of plots in each group was clearly different (Figure 4(a)). X-ray scores in the ONO-5334 group were distributed in the lower region compared to those in the control group, although joint swelling scores did not decrease. In the MTX group, both joint swelling and X-ray scores decreased, and in the combination group X-ray scores and not the joint swelling scores further decreased compared with the MTX group (Figure 4(b)).

### 3.5. Bone and Cartilage Turnover Markers

**Figure 5:**
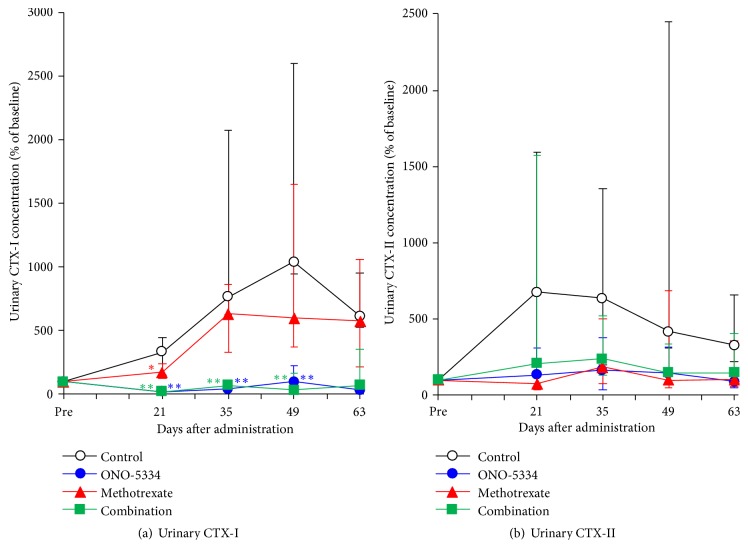
Percent changes in urinary CTX-I (a) and CTX-II (b) in collagen-induced arthritis monkeys. Data are expressed as median (quantile 3-1); control (n=8), ONO-5334 (n=9), methotrexate (n=8), and combination (n=9). *∗*p<0.05 and *∗∗*p<0.01 versus control group (Tukey test).

The bone resorption marker, urinary CTX-I in the control group, was markedly increased by sensitization, with a median change of approximately 10-fold at 7 weeks after the first sensitization compared with the levels before sensitization and then decreased gradually to 6-fold until the end of experimental period (Figure 5(a)). In the ONO-5334 and combination groups, concentrations of urinary CTX-I were maintained near the level before sensitization and significant at 3, 5, and 7 weeks after the first sensitization (p<0.01 vs the control group). MTX significantly decreased CTX-I at 3 weeks (p<0.05 vs the control group), whereas urinary CTX-I levels in the MTX group were roughly comparable with the control group from 5 weeks after dosing. The cartilage degradation marker, urinary CTX-II in the control group, increased approximately 7-fold at 3 weeks after the first sensitization compared with the level before sensitization and then decreased gradually to 3-fold until the end of experimental period (Figure 5(b)). ONO-5334, MTX, and combination treatments prevented this increase in CTX-II during the dosing period. However, there were no statistically significant differences in CTX-II among treatment groups.

## 4. Discussion

For the treatment of RA, it is important to prevent joint destruction to maintain physiological function. Cathepsin K is highly expressed in osteoclast and plays a crucial role for bone resorption [8–10, 14]. It is also expressed in the synovial tissue of RA patients and could be related to joint destruction [26, 27]. Therefore, this study assessed the preventive effects of ONO-5334 alone and in combination with MTX on joint destruction in the monkey collagen-induced arthritis model to determine its therapeutic potential in RA. ONO-5334 inhibited the progression of joint destruction and the effect was enhanced by concomitant use of ONO-5334 with MTX compared to MTX alone in this model.

Female monkeys were used here as they are phylogenetically and physiologically close to human beings [22] and because the incidence of arthritis is higher in women than in men. Although the rodent CIA model is widely used to evaluate antirheumatic drugs and the analysis of RA pathogenesis, there is a significant species difference in the way the immune system functions between human beings and rodents [28]. In rodent CIA models, joint inflammation usually occurs in the ankles; however, phalangeal joints become swollen in the monkey CIA model like RA patients. Furthermore, the role of cathepsin K in bone and cartilage metabolism may also be slightly different between rodents and primates. In humans, it has been shown that cathepsin K deficiency [29] and cathepsin K inhibitors led to decrease in CTX-I [20, 21, 30]. In contrast, both CTX-I and CTX-II have been shown to be elevated in cathepsin K deficient mice [31, 32]. However, cathepsin K inhibitors decreased serum and urine CTX-I in cynomolgus monkeys [18, 33]. The use of the monkey CIA model could help bridge the gap between rodent models and human patients, and as far as we know this is the first study investigating the effect of cathepsin K inhibitors in the monkey CIA model.

In this study, ONO-5334 alone showed no suppressive effect on joint swelling. It has been previously reported that cathepsin K inhibitors partially suppressed inflammation in adjuvant-induced arthritis (AIA) rats [34] and CIA mice [16]. Although it is not clear why cathepsin K inhibitors show anti-inflammatory effects in rodents, it has been reported that a cathepsin K inhibitor, NC-2300, decreased IL-12 production via inhibition of the Toll-Like Receptor-9 (TLR-9) signalling pathway [34]. We have previously investigated whether ONO-5334 has a similar effect to NC-2300 using human and mouse dendritic cells. We found in mouse dendritic cells that ONO-5334 significantly inhibited IL-12 production induced by the TLR-9 ligand CpG [35]. However, in human dendritic cells, the TLR-9 signalling pathway was not inhibited by ONO-5334 [36]. These results suggest that cathepsin K does not play an important role during the progress of inflammation in the monkey CIA model and that the anti-inflammatory effects of cathepsin K inhibitors in rodents may be different from those in primates.

X-ray examinations showed that ONO-5334 and ONO-5334/MTX treatment decreased joint space narrowing and/or bone atrophy and bone erosion or architectural joint destruction compared with control group. Following treatment with ONO-5334 or ONO-5334/MTX, concentrations of CTX-I and CTX-II were close to or below baseline. Since the anti-inflammatory effect of ONO-5334 was not observed in this model, it is suggested that ONO-5334 prevents joint destruction by inhibiting bone and cartilage destruction directly.

Concomitant use of ONO-5334 with MTX reduced joint swelling scores compared with ONO-5334 alone. This further reduction is considered to be attributable to MTX since the efficacy of the MTX and ONO-5334/MTX treatments on joint swelling was similar. X-ray scores in the ONO-5334/MTX group were lower than those in the MTX group. It is therefore possible that the effect of MTX on joint destruction could be augmented by administration in combination with ONO-5334. To further examine the effects of ONO-5334 alone and in combination with MTX on joint destruction, the relationship between joint swelling score and X-ray findings was explored. A scatter plot of the control group showed a linear relationship between joint swelling score and X-ray score and suggests that progression of joint destruction was proportional to increased arthritis severity. A scatter plot in the MTX group was aligned with that in the control group, which indicates that MTX suppresses swelling and then inhibits joint destruction. In contrast, ONO-5334 prevented joint destruction without showing anti-inflammatory effects. Thus, the distribution pattern in the ONO-5334 group was different from both control and MTX groups. This difference could be attributed to the different mechanisms of action of ONO-5334 and MTX.

It has been reported that Denosumab, an anti-RANKL antibody, significantly reduced bone erosion and CTX-I in RA patients compared with placebo [37]. This indicates that suppression of bone resorption may help to prevent joint destruction in RA patients. However, joint space narrowing score, an index of cartilage destruction, was not improved by Denosumab and this might be due to a lack of CTX-II suppression. Type II collagen is one of the major components of the extracellular matrix of cartilage and it is known to be cleaved by cathepsin K [7, 38]. Cathepsin K is thought to play a role in the destruction of articular cartilage in inflammatory arthritis. Interestingly, urinary CTX-II was significantly decreased with ONO-5334 in postmenopausal women [20]; ONO-5334 could potentially prevent cartilage destruction in patients with RA. Since there was no significant safety implications in the Phase II study for osteoporosis [21], ONO-5334 could be a safer and more convenient drug for the prevention of joint destruction in RA patients compared to biologics.

A possible limitation of this study was that there was no statistical significance in any parameters other than X-ray score and urinary CTX-I due to considerable variability in inducing arthritis in every animal. It has been reported that incidence of arthritis is 100% in the rodent CIA model and 60% in the monkey CIA model [22]. Another possible limitation was that the severity of disease in this arthritis model is considered to be higher than that of RA patients. Urinary CTX-I and CTX-II were increased 10- and 7-fold at their peak due to CIA in this model, respectively. In patients with RA, urinary CTX-II increased approximately 2-fold compared with healthy controls [39]. Furthermore, joint destruction was observed in only 9 weeks in this study, which is much faster than in the clinical condition.

## 5. Conclusions

In summary, inhibition of cathepsin K by ONO-5334 inhibited joint space narrowing, bone atrophy, bone erosion, and architectural joint destruction in this cynomolgus monkey arthritis model. However, it should be noted that ONO-5334 had no anti-inflammatory effects. We found that concomitant use of ONO-5334 with MTX reduced architectural joint destruction and bone resorption marker, CTX-I, compared to MTX alone. Therefore, ONO-5334 may help to prevent joint destruction in combination with MTX for the treatment of RA.

## Figures and Tables

**Table 1:** General condition score in collagen-induced arthritis monkeys.

**Treatment Group **	**Dosing (Day)**				
	**Pre-dose**	**21**	**35**	**49**	**63**

Control	0.0 ± 0.0	0.4 ± 0.3	1.1 ± 0.4	1.3 ± 0.5	1.3 ± 0.4
ONO-5334	0.0 ± 0.0	0.1 ± 0.1	0.7 ± 0.2	0.6 ± 0.2	0.4 ± 0.2
MTX	0.0 ± 0.0	0.1 ± 0.1	0.4 ± 0.3	0.4 ± 0.4	0.5 ± 0.5
ONO-5334/MTX	0.0 ± 0.0	0.2 ± 0.1	0.7 ± 0.1	0.9 ± 0.4	0.7 ± 0.3

Data are presented as mean ± SE; control (n=8), ONO-5334 (n=9), methotrexate (n=8), and combination (n=9). The general condition of animals was evaluated in accordance with the following criteria: score 0: no abnormality; score 1: difficulty in hanging from the bars of the home cage by the fingers; score 2: inability to hang from the bars of the home cage by the fingers (using wrist); score 3: movement only by using forelimbs or hindlimbs; score 4: crouching.

## Data Availability

The data used to support the findings of this study are available from the corresponding author on reasonable request.
